# Metabolomics of Dietary Fatty Acid Restriction in Patients with Phenylketonuria

**DOI:** 10.1371/journal.pone.0043021

**Published:** 2012-08-13

**Authors:** Ulrike Mütze, Skadi Beblo, Linda Kortz, Claudia Matthies, Berthold Koletzko, Mathias Bruegel, Carmen Rohde, Joachim Thiery, Wieland Kiess, Uta Ceglarek

**Affiliations:** 1 Department of Women and Child Health, Hospital for Children and Adolescents, University Hospital, University of Leipzig, Leipzig, Germany; 2 Clinical Chemistry and Molecular Diagnostics, Institute of Laboratory Medicine, University Hospital, University of Leipzig, Leipzig, Germany; 3 LIFE – Leipzig Research Center for Civilization Diseases, University Leipzig, Leipzig, Germany; 4 Division Metabolic Diseases and Nutritional Medicine, Dr. von Hauner Children’s Hospital, Ludwig-Maximilians-University of Munich, Munich, Germany; 5 Institute of Laboratory Medicine, Ludwig-Maximilians-University of Munich, Munich, Germany; Wageningen University, The Netherlands

## Abstract

**Background:**

Patients with phenylketonuria (PKU) have to follow a lifelong phenylalanine restricted diet. This type of diet markedly reduces the intake of saturated and unsaturated fatty acids especially long chain polyunsaturated fatty acids (LC-PUFA). Long-chain saturated fatty acids are substrates of mitochondrial fatty acid oxidation for acetyl-CoA production. LC-PUFA are discussed to affect inflammatory and haemostaseological processes in health and disease. The influence of the long term PKU diet on fatty acid metabolism with a special focus on platelet eicosanoid metabolism has been investigated in the study presented here.

**Methodology/Principal Findings:**

12 children with PKU under good metabolic control and 8 healthy controls were included. Activated fatty acids (acylcarnitines C6–C18) in dried blood and the cholesterol metabolism in serum were analyzed by liquid chromatographic tandem mass spectrometry (LC-MS/MS). Fatty acid composition of plasma glycerophospholipids was determined by gas chromatography. LC-PUFA metabolites were analyzed in supernatants by LC-MS/MS before and after platelet activation and aggregation using a standardized protocol. Patients with PKU had significantly lower free carnitine and lower activated fatty acids in dried blood compared to controls. Phytosterols as marker of cholesterol (re-) absorption were not influenced by the dietary fatty acid restriction. Fatty acid composition in glycerophospholipids was comparable to that of healthy controls. However, patients with PKU showed significantly increased concentrations of y-linolenic acid (C18:3n-6) a precursor of arachidonic acid. In the PKU patients significantly higher platelet counts were observed. After activation with collagen platelet aggregation and thromboxane B_2_ and thromboxane B_3_ release did not differ from that of healthy controls.

**Conclusion/Significance:**

Long-term dietary fatty acid restriction influenced the intermediates of mitochondrial beta-oxidation. No functional influence on unsaturated fatty acid metabolism and platelet aggregation in patients with PKU was detected.

## Introduction

Phenylketonuria (PKU; OMIM 261600) is one of the most common inborn metabolic diseases, approximately affecting one in 8000 newborns in Western Europe [1]. It is caused by deficient activity of phenylalanine hydroxylase. Children with untreated PKU suffer from severe physical and mental disability. Postnatal diagnosis by newborn screening, early treatment with a lifelong protein restricted diet and substitution of a phenylalanine free amino acid formula result in a normal cognitive development [2]. Since protein-rich foods such as meat, milk products, eggs, and fish are the predominant nutritional sources of animal fat, especially of saturated fatty acids and long chain polyunsaturated fatty acids (LC-PUFA), patients with PKU have a low dietary LC-PUFA intake [3], [4]. Reduced LC-PUFA concentrations in plasma and erythrocytes have been found previously [4]–[7]. LC-PUFA, as arachidonic acid (AA; 20:4n-6), docosahexaenoic acid (DHA; 22:6n-3) and eicosapentaenoic acid (EPA; 20:5n-3), are discussed to affect inflammatory and haemostaseological processes in health and disease [8]–[11]. AA, the precursor of eicosanoids, plays a central role in proinflammatory response and haemostasis [8]–[10].

There is some evidence that the reduced LC-PUFA intake may influence hemostaseological processes in PKU-patients. Decreased concentrations of AA and its metabolite, thromboxane B_2_ (TXB_2_), have been found in serum [5]. In addition, the low nutritional intake of saturated fatty acids seems to influence the concentrations of intermediates (acylcarnitines) of mitochondrial beta-oxidation of fatty acids. Patients with PKU showed a lower concentration of free carnitine (C0) [12]–[15] and lower concentrations of the activated fatty acids (acylcarnitines) octanoylcarntine (C8) and decanoylcarnitine (C10) in dried whole blood compared to controls [15]. Furthermore, cholesterol concentrations in plasma are described to be lower in patients with PKU under good metabolic control [16]–[18].

We studied the metabolic pathways of saturated and unsaturated fatty acids in patients with PKU and in controls, because these pathways are suspected, to be influenced by the long-term strict PKU diet. The aim was to get an overview of long term fatty acid metabolome in children with PKU under strict diet and to investigate for the first time the possible functional influences on homeostasis using a standardized non-invasive cell-model.

## Materials and Methods

### Participants

Patients with diagnosed classical PKU were eligible for inclusion if they were under good metabolic control; i.e. a documented average phenylalanine concentration in dried blood spots was below 360 µmol/L for patients <10 years, below 900 µmol/l for patients >10 years, determined at least monthly over the previous six months. Patients with PKU followed their usual treatment consisting of protein-restricted diet and supplementation of a phenylalanine free amino acid formula containing also vitamins and trace elements. Prior study entry, a diet record over three days was performed.

Healthy controls were recruited before elective surgery (n = 4, remove of orthopedic material) or before endocrinological testing (n = 4, arginine test, only patients with normal test results were included) and if they followed an omnivore Western diet. No patient or volunteer received additional medication; especially no cyclooxygenase inhibitors during the last 14 days prior study entry, or over the counter nutritional supplements. Study samples were collected while blood withdrawal for diagnostic reasons after fasting for 4 to 6 hours.

For standardization of platelet activation experiments, 10 healthy adult volunteers (age 23–37 years, 5 females, 5 males) were included after informed consent. All of them were on a omnivore Western diet, without medication, especially no cyclooxygenase inhibitors during the last 14 days prior blood withdrawal.

Blood (10 ml) was drawn without occluding the vein from each participant using multifly needle sets and polypropylene monovettes with and without anticoagulants (Ethylenediaminetetraacetic acid (EDTA), citrate and lithium-heparinate) obtained from Sarstedt (Nümbrecht, Germany). EDTA whole blood was dropped on filter paper (grade 903; GE Health Care, Germany).

### Procedures and Investigations

#### Baseline-parameters - clinical chemistry and routine analysis

Blood count parameters were measured on the Sysmex XE 2100 (Norderstedt, Germany). Coagulation parameters (activated partial thromboplastin time (aPTT), prothrombin time, fibrinogen, and antithrombin III (AT III) were measured on the BCS System (Siemens, Munich, Germany). Alanine aminotransferase, aspartate aminotransferase, gamma-glutamyl transferase, triglycerides, cholesterol, free fatty acids, creatinine, albumin, serum proteins, c-reactive protein, sodium, potassium, and calcium, folic acid, vitamin B12, and ferritin in serum were measured on the Modular Analytics system from Roche (Mannheim, Germany). Amino acids in plasma were analyzed by ion exchange chromatography (IEC) and ninhydrine derivatisation.

#### Analysis of acylcarnitines in dried blood

Acylcarnitines in dried blood spots were measured as formerly described by liquid chromatography/tandem mass spectrometry (LC-MS/MS) [19]. Briefly, 3.0 mm diameter punches (containing approx. 3 µL) of the dropped-on filter paper were placed into 96-well polypropylene micro titer plates and extracted with 100 µL of a methanol solution, containing isotope labelled amino acid and acylcarnitine internal standards (NSK-A, NSK-B, Cambridge Isotope Laboratories, Andover, USA), for 30 min by gentle shaking at room temperature. After evaporation at 70°C for 40 min, 60 µL of 3n butanolic-HCL was added for derivatisation at 65°C. After a second evaporation step the samples were reconstituted with 150 µL of the mobile phase (1/1 v/v isopropanol/water) and analysed by flow injection analysis (FIA)-MS/MS using a API 2000 triple quadrupole mass spectrometer (AB SCIEX, Darmstadt, Germany).

#### Analysis of sterols in serum

Serum concentrations of brassicasterol, campesterol, stigmasterol, beta-sitosterol, cholesterol, lanosterol and desmosterol were measured by our formerly described LC-MS/MS method using a API 3000 triple quadrupole mass spectrometer with photospray ionization (AB SCIEX, Darmstadt, Germany) [20]. 10 µL of human serum were diluted with 490 µL of 50/50 v/v methanol/acetonitrile. After centrifugation, free and esterified beta-sitosterol, campesterol, and brassicasterol were simultaneously analyzed by LC-MS/MS.

#### Fatty acid composition of plasma glycerophospholipids

The analysis of plasma glycerophospholipid composition was performed by a sensitive and precise high-throughput method recently described [21]. In brief, 100 µl of serum, 100 µl of internal standard (1,2-dipentadecanoyl-sn-glycero-3-phosphocholine dissolved in methanol) and 0.6 ml methanol (precooled to 5°C) were combined in glass tubes and shaken for 30 s. Samples were centrifuged and the supernatant was transferred into another glass tube. Sodium methoxide solution (25 µl) was added, tubes were shaken, and synthesis of methyl esters proceeded at room temperature. The reaction was stopped after 3 minutes by adding 75 µl methanolic HCl. Fatty acid methyl ester (FAME) were extracted twice by adding 300 µl hexane at a time. Extracts were combined and dried under nitrogen flow at room temperature and taken up in 50 µl hexane (containing 2 g/l BHT) for gas chromatographic (GC) analysis. Individual FAMEs were quantified by GC with flame ionization.

#### Eicosanoid analysis in plasma and supernatants

Eicosanoids in EDTA plasma and supernatants after activation of platelet rich citrate-plasma were analyzed by LC-MS/MS [22]. Solid phase extraction (SPE) was used for extraction and concentration of the eicosanoids [23]. 150 µl of the platelet supernatants or EDTA plasma were spiked with 50 µl 1/1 v/v methanol/water containing 0.2 µg/ml of arachidonic acid-d8 (AA-d8) and 0.02 µg/ml of prostaglandin F_2α_-d4 (PGF_2α_-d4), prostaglandin E_2_-d4 (PGE_2_-d4), leukotriene B_4_-d4 (LTB_4_-d4), and 5-S-hydroxyeicosatetraenoic acid-d8 (5-HETE-d8), thromboxane B_2_-d4 (TxB_2_-d4), eicosapentaenoic acid-d5 (EPA-d5), and docosahexaenoic acid-d5 (DHA-d5). The samples were extracted using Strata X SPE columns (Phenomenex, Aschaffenburg, Germany). Samples were dried under nitrogen and stored at −80°C until analysis. After elution 10 µl of each aliquot were injected into the LC-MS/MS system. Chromatographic separation was performed based on the protocols by Deems et al [23]. A triple quadrupole mass spectrometer (API 5500 QTrap, SCIEX Toronto, Canada) using electrospray ionization (ESI) was used in negative ion mode. Analytes were quantified in multiple reaction monitoring transitions (MRMs) using the corresponding labeled internal standards [22]. Table S1 shows the analyzed metabolites.

#### Platelet function analysis

In a first step a standardized protocol for reproducible platelet activation with collagen and analysis of subsequent eicosanoid release was developed.

For standardization TXB_2_ release after platelet activation was used. The measured metabolite TXB_2_ is a stable, biologically inert metabolite formed from the non-enzymatic hydrolysis of TXA_2_ [24]. The latter has a half-life of approximately 30 seconds [24]. TXB_2_ in plasma and urine reflects platelet TXA_2_ synthesis [25]. Thromboxane B_3_ (TXB_3_) is the equivalent metabolite metabolized competitively by the same enzymes from omega-3 LC-PUFA (i.e. EPA) [8].

Platelet-rich plasma (150 Gpt/l ±15) of freshly drawn citrate-blood from five healthy adult volunteers was activated with collagen. After 10 minutes of platelet activation (compared to 1 and 5 minutes) with collagen a plateau of TXB_2_ release was found. Variability for TXB_2_ release after platelet activation with collagen was tested. Intra-assay variability, activation three times consecutively, was ≤6.2%. Between-day variability, at three consecutive days, blood withdrawal at noon, was ≤8.7%. Circadian variability was ≤14.8%. Therefore, platelet activation from five healthy subjects was performed after blood withdrawal once in the morning, at noon and in the afternoon at three consecutive days. Interindividual variability of TXB_2_ release after platelet activation with collagen was 15.6%.

From this data the following standardized protocol was established: 225 µl platelet-rich plasma (150 Gpt/l ±15 Gpt/l) from freshly drawn citrate-blood was transferred to three micro glass silicon tubes with magnetic stirrers on Platelet Aggregation Profiler 4 (PAP 4, mölab, Langenfeld, Germany). In two samples platelet activation was performed with bovine collagen (25 µl, 1.9 g/l) for 10 minutes, registered by the PAP 4 (mölab, Langenfeld, Germany). The third sample remained inactivated for 10 minutes. Afterwards, stirrers were removed, plasma transferred to 0.5 ml tubes (Eppendorf, Hamburg, Germany) and centrifuged at 5260 g for 10 minutes (20°C) to remove platelets and cell detritus. The supernatants were stored at −80°C under nitrogen to avoid oxygen-induced radical formation until analysis.

### Ethics

The study protocol was approved by the local ethics committee of the Medical Faculty of the University of Leipzig (Leipzig, Germany; registration number 021-09-180808). The trial was performed in accordance with the Declaration of Helsinki/Somerset West and followed ICH-GCP guidelines. Informed written consent was obtained from all patients and/or parents.

### Statistical Methods

All statistical analyses were performed using PASW statistic 18 (Chicago, IL, USA). For descriptive reasons we displayed the median and minimum/maximum of all data. Wilcoxon-test, for paired samples, or Mann-Whitney-U-test, for independent samples, was used. A probability value of p<0.05 was considered significant.

For initial analysis the study population was divided in groups regarding age (patients <10 years, patients > = 10 years) or regarding diet (phenylalanine consumption <300 mg/day, 300–400 mg/day, >400 mg/day). This did not influence the results. Therefore, final analysis was performed using the entire sample.

## Results

### Participants

12 (6 females, 6 males) early treated children and adolescents with classical PKU aged 5–14 years and 8 healthy controls (5 females, 3 males) aged 5–17 years were included in the study.

Age, height, weight, body mass index (BMI) and Tanner stages did not differ significantly between the two groups (Table S2).

### Diet Records and Basic Laboratory Parameters

The results of the diet records of patients with PKU were compared to the regular daily allowance, recommended for the German speaking countries [26] as shown in table 1. The overall energy intake was just below the recommended amount. None of the 12 PKU-patients had signs of over- or undernutrition with a median BMI SDS of 0.6 (−1.24–1.51; Table S2). Nevertheless, the patients reached only about 75% of the recommended fat intake under the strict PKU diet. Median fat intake from vegetable sources was 56.9% (45.1–91.8%; predominant vegetable oil) and from animal sources was 42.7% (8.2–55.1%, predominant butter). Although fat intake was under the recommended amount, the precursors of AA and DHA, linoleic acid and alpha-linolenic acid, respectively, were consumed as recommended (Table 1).

**Table 1:** Analysis of three-day diet record of patients with phenylketonuria (PKU).

	Patients with PKU(n = 12)median (range)
Energy intake [kcal/day]	1678.0 (919–2075)
% DACH RDA	98 (66–131)
Carbohydrates [g/day]	232.6 (148.4–367.5)
% DACH RDA	93 (69–127)
Proteine [g/day]	41.1 (22.5–78.3)
% DACH RDA	197 (132–321)
Fat [g/day]	47.3 (18–65)
% DACH RDA	75 (28–125)
Phenylalanine [mg/day]	338 (254–690)
Linoleic acid [mg/day]	4536 (876–10866)
% DACH RDA	110 (67–240)
alpha-linolenic acid [mg/day]	805 (219–1461)
% DACH RDA	96 (22–182)

% DACH RDA: percentage of regular daily allowance, recommended for the German speaking countries [26].

Carbohydrate intake was slightly below the recommended amount and the intake of protein was sufficient under substitution of amino acid formula [26] (Table 1).

Basic liver and kidney parameters, electrolytes, lipids, infection parameters, and coagulation parameters were within the normal range in both groups without significant differences. In PKU patients, essential amino acid concentrations in plasma were within the normal range except from the elevated phenylalanine concentrations (Table S2).

**Figure 1:**
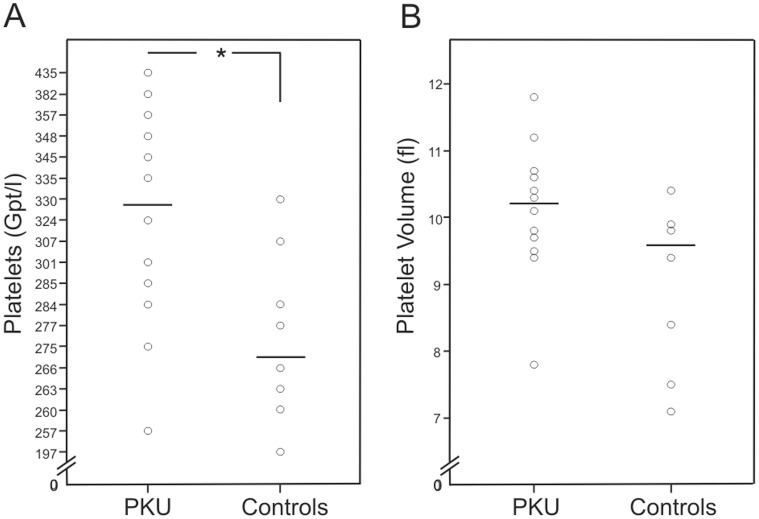
Platelet count and platelet volume. Data of patients with phenylketonuria (PKU) (n = 12) and healthy controls (n = 8). **A** platelet count (* p<0.05) and **B** platelet volume. The stripe marks the median.

Patients with PKU showed a significant higher median platelet count (329 Gpt/l, range 257–435 Gpt/l) compared to controls (271.5 Gpt/l, range 197–330 Gpt/l) (p<0.05). Platelet volume in patients with PKU was 10.2 fL (7.8–11.8 fL) compared to controls 9.6 fL (7.1–10.4 fL) (p = 0.063) (Figure 1, Table S2). Plasma concentrations of folic acid (p<0.01), vitamin B12 (p<0.01), and ferritin (p<0.05) in patients with PKU were significantly higher than in controls (Table S2).

### Investigations of Saturated Fatty Acid Metabolism, Mitochondrial Beta-oxidation, Cholesterol Adsorption and Synthesis

**Figure 2:**
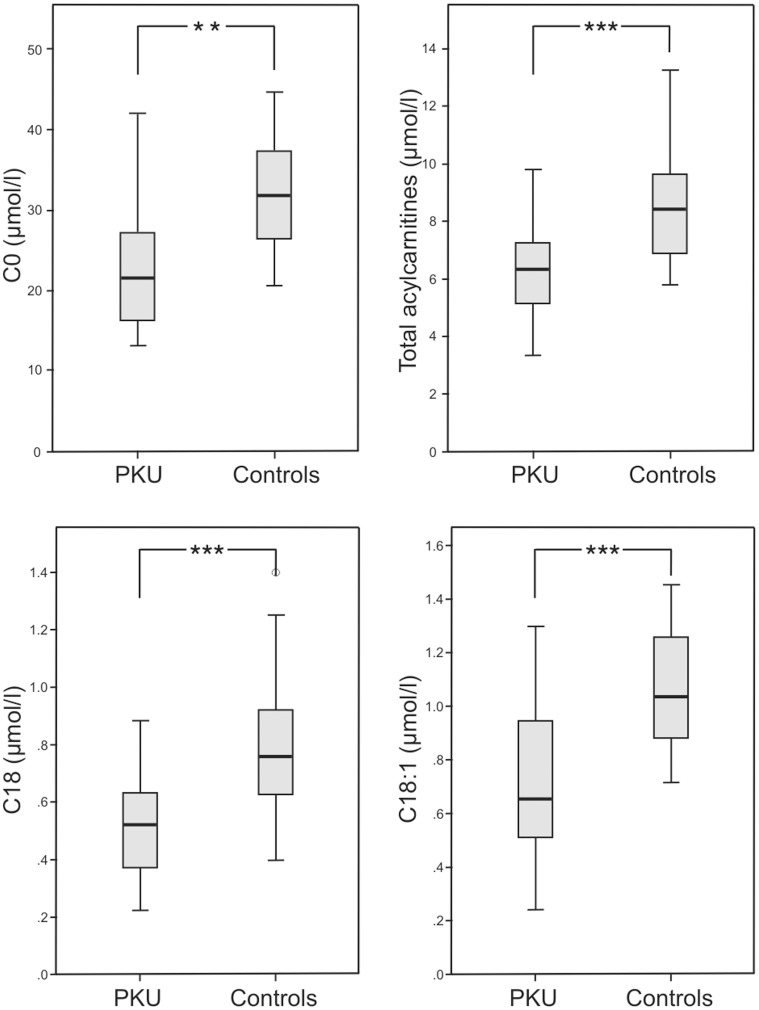
Free carnitine and acylcarnitines. Data of patients with phenylketonuria (PKU) (n = 12) and healthy controls (n = 8); measured by liquid chromatography/tandem mass spectrometry (LC-MS/MS). **A** Free carnitine (C0) in dried blood (**p<0.01). **B** Total acylcarnitines in dried blood (***p<0.001). **C** Octadecanoylcarnitine (C18) in dried blood (***p<0.001). **D** Octadecenoylcarnitine (C18:1) in dried blood (***p<0.001).

Patients with PKU had significantly lower concentrations of free carnitine (C0) (p<0.01), total acylcarnitines (p<0.001) and of activated fatty acids of the different chain-lengths (p<0.001: C2, C3, C18, C18OH, C18:1), (p<0.01: C4–0H, C5, C8), (p<0.05: C6, C10:1, C14:1, C16:1, C18:2) compared to the healthy controls (Figure 2, Table S3).

Serum concentrations of cholesterol absorption markers brassicasterol, campesterol, stigmasterol, beta-sitosterol, and lanosterol as marker for cholesterol synthesis did not differ significantly between both groups. Also the cholesterol concentration and the relative non-cholesterol concentrations showed no significant differences (Table S3).

### Investigation of Unsaturated Fatty Acid Metabolism

**Figure 3:**
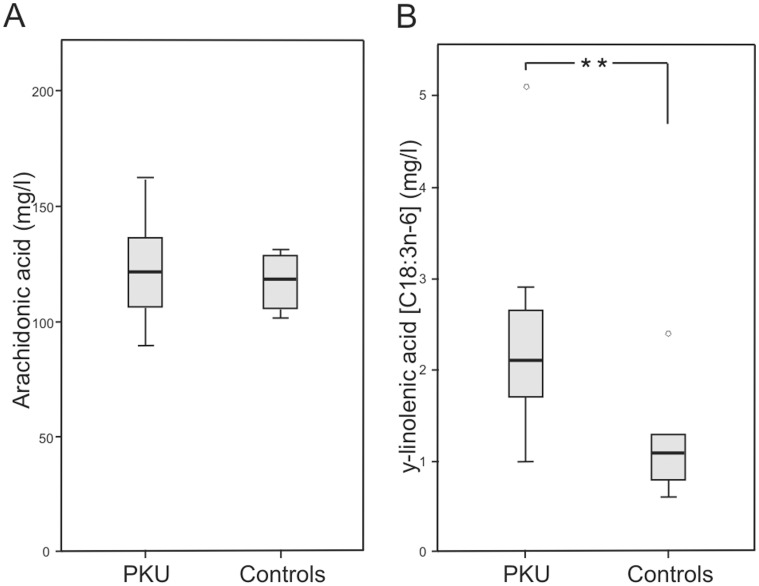
Arachidonic acid and y-linolenic acid concentrations in plasma glycerophospholipids. Data of patients with phenylketonuria (PKU) (n = 12) and healthy controls (n = 8). **A** Arachidonic acid in plasma glycerophospholipids. **B** y-linolenic acid in plasma gylcerophospholipis (** p<0.01).

Patients with PKU showed significant higher concentrations of y-linolenic acid (C18:3n-6) (2.1 mg/l, range 1.0–5.1 mg/l versus 1.1 mg/l, range 0.6–2.4 mg/l; p<0.01) and a trend to higher concentrations of di-homo-y-linolenic acid (C20:3n-6) (43.3 mg/l, range 33.0–60.0 mg/l versus 36.2 mg/l, range 30.0–52.8 mg/l; p = 0.069) in plasma glycerophospholipids (Table S3, Figure 3). Both are precursors in endogenous AA synthesis. For other fatty acids of plasma glycerophospholipids no differences between patients with PKU and controls were found (Table S3).

There was no difference for TXB_2_ (derived from AA) plasma concentrations in the study groups. However, TXB_3_ (derived from EPA) plasma concentrations were significantly lower in patients with PKU (0.01 ng/ml, range 0.00–0.12 ng/ml) compared to controls (0.10 ng/ml, range 0.03–0.25 ng/ml; p<0.005; Table S3).

### Functional Investigations of Platelet Activation Induced Eicosanoid Release

Platelet activation with collagen for 10 minutes induced a significant release of AA, TXB_2_, TXB_3_, prostaglandin E_2_ (PGE_2_), prostaglandin D_2_ (PGD_2_), 11-S-hydroxyeicosatetraenoic acid (11-HETE), and 12-S-hydroxyeicosatetraenoic acid (12-HETE) in patients with PKU and controls (Table 2). The concentrations of TXB_2_, TXB_3_, PGE_2_, PGD_2_, 11-HETE, and 12-HETE after platelet activation with/without collagen did not differ significantly between the two groups. To assure reproducibility of analysis of eicosanoid release after platelet activation we validated a standardized protocol for the activation experiments.

**Table 2:** Eicosanoid concentrations in supernatants after 10 minutes of activation of platelet rich plasma (225 µl; 150±15 Gpt/l) with/without collagen in patients with phenylketonuria (PKU) and healty controls.

	PKU (n = 11)median (Range)		Controls (n = 8)median (Range)	
	Without collagen	With collagen	Without collagen	With collagen
AA [ng/ml]	1470 (948–2470)	3720 (2880–5520)	1520 (1070–2610)	3830 (3400–5130)
TXB2 [ng/ml]	6.0 (2.7–21.8)	390 (252–502)	3.9 (2.2–10.2)	351 (262–534)
TXB3 [ng/ml]	0.17 (0–0.46)	2.56 (0.77–4.28)	0.1 (0–0.4)	2.59 (1.57–3.59)
PGE2 [ng/ml]	0.24 (0.1–2.65)	11.5 (6.0–15.5)	0.20 (0–0.33)	11.2 (5.9–17.6)
PGD2 [ng/ml]	0.08 (0.02–0.24)	1.56 (0.51–4.38)	0.03. (0.01–0.08)	1.0 (0.51–1.97)
11-HETE [ng/ml]	1.64 (0.84–2.36)	44.4 (26.2–58)	1.45 (0.96–3.14)	38.3 (29.6–366)
12-HETE [ng/ml]	9.03 (4.18–15.6)	299 (120–425)	7.37 (3.71–11.5)	279 (209–411)

AA = arachidonic acid, TXB_2_, = thromboxane B_2_, TXB_3_ = thromboxane B_3_, PGE_2_ = prostaglandin E_2_, PGD_2_ = prostaglandin D_2_, 11-HETE = 11-S-hydroxyeicosatetraenoic acid, 12-HETE = and 12-S-hydroxyeicosatetraenoic acid.

## Discussion

PKU-patients have to undergo a livelong phenylalanine restricted diet beginning in the first weeks of life, which is associated with a long-term restriction of saturated and nonsaturated fatty acids compared to a regular western diet [5], [7], [13]–[15], [17], [18], [27].

As may be expected, the investigated PKU patients had only 75% (median 47,3 g per day) of the age related recommended fat intake [26]. Similar results have been found previously [18], [27], [28]. Moreover, an inverse relation of animal to vegetable fat was observed in our PKU group (42.7% animal fat/56.9% vegetable fat). In contrast healthy German children 6–11 years/12–17 years consume 62.5 g/100.5 g fat per day, including 65.7% animal fat and 34.3% vegetable fat [29]. However, the intake of the essential fatty acids linoleic acid and alpha-linolenic acid, precursors for endogenous AA and DHA synthesis, was in the recommended range [26].

The influence of the restricted intake of saturated fatty acids with the PKU diet was found to be evident in both, the metabolism of C0 and the intermediates of mitochondrial ß-oxidation [15]. We observed significantly lower C0 and acylcarnitine concentrations in dried whole blood of PKU patients, although free fatty acids in serum were comparable between PKU patients and controls. Lower C0 concentrations in patients with PKU on a strict diet have been described previously as possible consequence of the low nutritional carnitine intake [14] or the reduced endogenous synthesis due to the lower plasma concentrations of the essential substrates methionine and lysine [15]. Additionally phenylacetic acid, a pathological metabolite found in serum and urine of PKU-patients, was found to be capable of inhibiting the endogenous carnitine synthesis [30]. However concentrations of C0 are not as low as in other inborn metabolic diseased like carnitine transporter defects known to show clinical symptoms, as (cardio-) myopathy. However, up to now, there is no data of long-term follow up in adult PKU patients available. Interestingly, carnitine substitution in amino acid formula appears not to affect carnitine and acylcarnitine status in patients with PKU [15]. We could confirm these findings, as the carnitine substitution (mean 27 mg/d, 10–48 mg/day per person) of four PKU patients of our study group did not affect their carnitine and acylcarnitine status (data not shown).

Next to reduced C8 und C10 concentrations in dried whole blood in PKU patients, as already being described in a previous study [15], also decreased concentrations of the C16- and C18-fatty acids as substrates of mitochondrial beta-oxidation as well as their intermediates (C14–C6) were found. The reduced concentrations of C18 acylcarnitine and of the intermediates may reflect the reduced fat intake, a diminished mobilization of fatty acids from fat tissue, and a lower energy production by ß-oxidation in patients with PKU. Furthermore: as result acetyl-CoA (measured as acetylcarnitine C2), the central metabolite in the energy metabolism and the link between carbohydrate, amino acid, and fatty acid metabolism, was significant lower in PKU patients compared to controls. However, no association to lower phospholipids, free fatty acids, ketone bodies, or cholesterol concentrations could be revealed.

Because of the lower total fat intake and the higher amount of nutritional fat intake as vegetable oil in the PKU diet [28], we initially suspected that serum cholesterol might be lower and the cholesterol synthesis might be higher in PKU patients compared to controls. In our study cohort, serum cholesterol concentrations did not differ between patients with PKU and healthy controls. These findings are in contrast to previous studies revealing lower serum cholesterol concentrations in patients with PKU [16], [17], [27], especially while under good metabolic control [18], and also compared to patients with a protein-restricted diet due to other inborn errors of amino acid metabolism [27]. Other studies showed a possible inhibition of cholesterol synthesis in vitro [31] and in animal models of PKU [32]. Colomé et al. [27] detected an inverse correlation of phenylalanine and cholesterol concentrations in serum and hypothesized high plasma phenylalanine concentrations to inhibit cholesterol synthesis. We could not confirm these results, because the marker for cholesterol synthesis lanosterol and the lanosterol/cholesterol ratio did not differ significantly between the two groups, who were comparable in BMI and Tanner stages.

LC-PUFA play an important role in neuronal development and cognitive function [33]–[37]. The imbalanced PKU diet with nearly absence of animal proteins and markedly reduced fatty acids of animal sources is known to result in lower plasma and cell membrane concentrations of LC-PUFA [4]–[7]. We could not confirm reduced LC-PUFA levels in the PKU study group. AA, EPA and DHA in plasma glycerophospholipids, a good biomarker for dietary fatty acid intake and body status [21], were comparable in both study groups and adequate regarding reference values of serum glycerophospholipids in children [38], [39]. This may be caused by a higher endogeneous LC-PUFA synthesis in our patients supported by the fact that we found higher concentrations of AA precursors y-linolenic acid (C18:3n-6) and di-homo-y-linolenic acid (C20:3n-6) in plasma glycerophospholipids. In addition, intake of the essential fatty acids linoleic acid and a-linolenic acid was as recommended. Former studies reported that even an enhanced dietary intake of a-linolenic acids was not sufficient to increase DHA-phosholipid concentrations in infants [40]–[42].

Reactive thrombocytosis was described to be associated with metabolic diseases in children [43], [44]. The PKU patients studied here had higher platelet count and a trend to higher platelet volume compared to controls, but showed no other signs leading to reactive thrombocytosis (e.g. no infection, iron, cobalamin, and folic acid deficiency). These results were reproduced in a bigger PKU cohort of our outpatient clinic (n = 49, data not shown).

Plasma AA and TXB_2_ concentrations did not differ between both groups. In contrast, Agostoni and colleagues found reduced serum TXB_2_ and AA concentrations [5]. However, plasma concentrations of TXB_3_, metabolite of EPA, were significantly lower in PKU patients compared to the control group. This may indicate a reduced omega-3 LC-PUFA metabolism in PKU patients, although, the amount of EPA and DHA in plasma glycerophospholipids was adequate regarding reference values [38], [39].

Based on the fact that we found more and larger platelets which are known to contain more granules producing higher amounts of vasocative and prothombotic factors [45]–[47] and the hypothesis that the dietary restriction of fatty acids, especially LC-PUFA, affects the platelet function, we established a well standardized protocol for platelet activation and eicosanoid release. Collagen was chosen for activation of the cyclooxygenase and lipoxygenase pathway. No differences between the two groups concerning aggregation and platelet eicosanoid release were found, neither analysing TXB_2,_ nor TXB_3_. The same was true for total eicosanoid concentrations with or without activation and for the percental increase of eicosanoid concentrations after activation. This is an important finding, as it shows that although PKU patients follow a strict diet, which influences LC-PUFA status, cellular eicosanoid metabolism seems not to be affected as it were shown for platelet aggregation. Nevertheless, we found more than 15% interindividual variability of TBX_2_ release. The number of study subjects in each group maybe therefore too small to answer this question definitely.

In conclusion, fat is an essential part of nutrition. It affects many different metabolic pathways. We applied a LC-MS/MS approach to some of these pathways to analyze the influence of the moderate long-term fatty acid restriction in PKU patients on the metabolome of saturated and unsaturated fatty acids and found for the first time the diet to influence the complete mitochondrial ß-oxidation pathway and energy metabolism, but not cholesterol metabolism. In our newly established cellular experiment, patients with PKU were found to have more large platelets, but the aggregation and platelet eicosanoid release was not different from healthy controls. To assess the value of these findings for the possible resulting comorbidities, the long-term outcome of PKU patients, and potential therapeutic approaches further studies with bigger cohorts and older PKU patients should be investigated.

## Supporting Information

Table S1 **Eicosanoids and long chain polyunsaturated fatty acids (LC-PUFA) measured in plasma by liquid chromatography/tandem mass spectrometry (LC-MS/MS)** [**22**] **.**
(DOC)

Table S2 **Baseline data of patients with phenylketonuria (PKU) (n = 12) and healthy controls (n = 8).**
(DOC)

Table S3 **Concentrations of activated fatty acids (acylcarnitines), sterols, and plasma glycerophospholipids composition in patients with phenylketonuria (PKU) and healthy controls.**
(DOC)
