# Supplementary material for: Metabolomics of Dietary Fatty Acid Restriction in Patients with Phenylketonuria
Source: PLoS One. 2012 Aug 13;7(8):e43021. doi: 10.1371/journal.pone.0043021 (PMC3418234; doi:10.1371/journal.pone.0043021)
Supplement: Table S1 — Eicosanoids and long chain polyunsaturated fatty acids (LC-PUFA) measured in plasma by liquid chromatography/tandem mass spectrometry (LC-MS/MS) [22] . (DOC) [file pone.0043021.s001.doc]

**Table S1**

| **Analyte** | **Abbrevation** | **Enzyme** |
| --- | --- | --- |
| Arachidonic acid | AA | Substrate |
| Prostaglandin F_2α_ | PGF_2α_ | Cyclooxygenase (COX) |
| Prostaglandin E_2_ | PGE_2_ | COX |
| Prostaglandin D_2_ | PGD_2_ | COX |
| Isoprostane F_2α_ | Iso-PGF_2α_ | Reactive oxigen species (ROX) |
| Thromboxane B_2_ | TXB_2_ | COX |
| Leukotriene B_4_ | LTB_4_ | Lipooxygenase (LOX) |
| 5-S-hydroxyeicosatetraenoic acid | 5-HETE | LOX |
| 11-S-hydroxyeicosatetraenoic acid | 11-HETE | LOX |
| 12-S-hydroxyeicosatetraenoic acid | 12-HETE | LOX |
| 5-oxo-Eicosatetraenoic acid | 5-oxo-ETE | LOX |
| 5-Hydroperoxyeicosatetraenoic acid | 5HpETE | LOX |
| 5,6-Dihydroxyeicosatetraenoic acid | 5,6-DHET | LOX |
| Lipoxin A4 | LXA4 | LOX |
| Docosahexaenoic acid | DHA | Substrate |
| Eicosapentaenoic acid | EPA | Substrate |
| Thromboxane B_3_ | TXB_3_ | COX |
