# Supplementary material for: Metabolomics of Dietary Fatty Acid Restriction in Patients with Phenylketonuria
Source: PLoS One. 2012 Aug 13;7(8):e43021. doi: 10.1371/journal.pone.0043021 (PMC3418234; doi:10.1371/journal.pone.0043021)
Supplement: Table S2 — Baseline data of patients with phenylketonuria (PKU) (n = 12) and healthy controls (n = 8). (DOC) [file pone.0043021.s002.doc]

**Table S2**

|  | **PKU (6m/6f)**  **median (range)** | | **Controls (3m/5f)**  **median (range)** | **p-value** |
| --- | --- | --- | --- | --- |
| **Clinical data** |  | |  |  |
| Age [years] | 7.88 (5-14) | | 9.75 (5-17) | 0,374 |
| Weight/SDS [kg] | 27.6/0.26 (19.2/ -1.2 -66.3/1.17) | | 28.4/ -0.43 (11.5/ -3.3 -64/1.15) | 0,939 |
| Height/SDS [cm] | 129.5/ 0.38 (112.8/-1.41-169/ 1.04) | | 132.2/-0.67 (93.4 /-357-169/1.78) | 0,671 |
| BMI/SDS [kg/m²] | 16.68/ 0.6 (14.51/ - 1.24 – 23.21/ 1.51) | | 16.22/ -0.1 (13.15/-1.71-22.41/1.18) | 0.487 |
| **Laboratory data** |  | |  |  |
| Alanine aminotransferase [ukat/l] | 0.27 (0.2.0.57) | | 0.25 (0.18-0.38) | 0.392 |
| Aspartate aminotransferase [ukat/l] | 0.59 (0.29-0.74) | | 0.51 (0.37-0.84) | 0.487 |
| Gamma-glutamyl transferase [ukat/l] | 0.2 (0.14-27) | | 0.17 (0.07-0.35) | 0.281 |
| Prothrombine time [%] | 97 (73-105) | | 95 (84-119) | 0.588 |
| Activated thromboplastin time [s] | 33.9 (29.7-38.4) | | 34 (31.4-36.9) | 0.939 |
| Fibrinogen [g/l] | 2.45 (2.1-4.3) | | 2.62 (2.1-3.5) | 0.414 |
| Antithrombin III [%] | 109 (98-116) | | 109 (96-118) | 0.934 |
| Cholesterol [mmol/l] | 4.1 (3.15-5.18) | | 3.83 (3.51-4.26) | 0.735 |
| HDL-cholesterol [mmol/l] | 1.15 (0.72-1.66) | | 1.25 (1.15-1.56) | 0.375 |
| LDL-cholesterol [mmol/l] | 2.14 (1.24-3.01) | | 2.09 (1.89-2.33) | 0.866 |
| Triglycerides [mmol/l] | 1.01 (0.69-1.81) | | 1.13 (0.58-1.79) | 0.552 |
| Free fatty acids [mmol/l] | 0.25 (12-58) | | 0.38 (22-83) | 0.239 |
| Creatinine [µmol/l] | 38.5 (30-45) | | 42 (29-53) | 0.670 |
| Sodium [mmol/l] | 140.7 (135.3-143.8) | | 140.2 (139.4-141.8) | 0.611 |
| Potassium [mmol/l] | 4.3 (3.9-4.9) | | 4.2 (4.1-4.5) | 0.495 |
| Calcium [mmol/l] | 2.4 (2.2-2.5) | | 2.4 (2.2-2.6) | 0.735 |
| Albumin [g/l] | 46.3 (44.1-50.9) | | 47 (43.8-50.7) | 0.499 |
| Total serum protein [g/l] | 70.5 (63.7- 73.0) | | 70.3 (66.4-74.4) | 0.933 |
| C-reactive protein [mg/l] | 0.4 (<0.3-7.2) | | 0.6 (<0.3-1.2) | 0.665 |
| Hemoglobin [mmol/l] | 7.9 (7.4-8.8) | | 7.7 (7.5-9.0) | 0.439 |
| Hematocrit | 0.37 (0.34-0.42) | 0.38 (0.35-0.42) | | 0.306 |
| Mean corpuscular volume (MCV) [fl] | 80.2 (75.9-89.8) | 82.2 (77.3-86.4) | | 0.728 |
| Mean corpuscular hemoglobin (MCH) [fmol] | 1.74 (1.61.1.87) | 1.68 (1.57-1.78) | | 0.131 |
| Platelets [Gpt/l ] | 329 (257-435) | 271.5 (197-330) | | **p<0.05** |
| Platelet volume [fl] | 10.2 (7.8-11-8) | 9.6 (7.1-10.4) | | 0.063 |
| Leucocytes [Gpt/l ] | 7.9 (4.8-11.1) | 6.6 (5.2-11.2) | | 0.280 |
| Folic acid [nmol/l] | >45.4 (30.3->45.4) | 28.1 (15.4-45.2) | | **p < 0.01** |
| Vitamine B12 [pmol/l] | 774.8 (289.9-1229) | 352.6 (238.6 -500) | | **p < 0.01** |
| Ferritin [ng/ml] | 29.67 (22.56-61.37) | 20.44 (17.97-36.84) | | **p < 0.05** |
| **Amino acids in plasma** |  |  | |  |
| Phosphoserine [µmol/l] | 1.0 (0-3) | 1.5 (1-2) | | 0.229 |
| Taurine [µmol/l] | 49.5 (32-61) | 55 (41-94) | | 0.069 |
| Phosphoethanolamine [µmol/l] | 0.5 (0-2) | 1 (0-1) | | 0.429 |
| Urea [µmol/l] | 4254 (2525-5274) | 4183 (2718-5525) | | 0.939 |
| Aspartic acid [µmol/l] | 4 (2-11) | 3 (0-18) | | 0.308 |
| Hydroxyproline [µmol/l] | 14 (0-26) | 32 (0-42) | | **<0.01** |
| Threonine [µmol/l] | 130 (89-218) | 109 (90-221) | | 0.418 |
| Serine [µmol/l] | 120 (82-180) | 132 (120-175) | | 0.142 |
| Asparagine [µmol/l] | 27 (18-44) | 48 (30-59) | | **<0.01** |
| Glutamic acid [µmol/l] | 41 (22-66) | 37 (24-81) | | 0.671 |
| Glutamine [µmol/l] | 553 (395-660) | 596 (513-756) | | 0.247 |
| Proline [µmol/l] | 215 (134-399) | 222 (70-327) | | 0.758 |
| Glycine [µmol/l] | 286 (212-434) | 251 (195-318) | | 0.097 |
| Alanine [µmol/l] | 415 (277-675) | 333 (190-473) | | 0.123 |
| Citrulline [µmol/l] | 27 (23-34) | 31 (24-47) | | 0.164 |
| Valine [µmol/l] | 266 (189-421) | 225 (154-332) | | 0.064 |
| Cystine [µmol/l] | 39 (23-66) | 45 (29-81) | | 0.28 |
| Cystathionine [µmol/l] | 0 (0-0) | 0 (0-1) | | 0.221 |
| Methionine [µmol/l] | 19.5 (10-34) | 20.5 (27-31) | | 0.462 |
| Isoleucine [µmol/l] | 75 (33-126) | 57 (31-95) | | 0.418 |
| Leucine [µmol/l] | 126 (60-222) | 124 (74-174) | | 0.908 |
| Tyrosine [µmol/l] | 74 (36-157) | 67 (45-88) | | 0.589 |
| Phenylalanine [µmol/l] | 302 (72-930) | 53 (48-75) | | **<0.001** |
| Histidine [µmol/l] | 77 (46-113) | 81 (71-96) | | 0.232 |
| Tryptophan [µmol/l] | 48 (35-116) | 49 (31-63) | | 0.537 |
| Ornithine [µmol/l] | 76 (56-121) | 71 (42-121) | | 0.417 |
| Lysine [µmol/l] | 184 (127-333) | 168 (136-277) | | 0.418 |
| Arginine [µmol/l] | 74 (33-150) | 75 (51-104) | | 0.396 |
