# Supplementary material for: Metabolomics of Dietary Fatty Acid Restriction in Patients with Phenylketonuria
Source: PLoS One. 2012 Aug 13;7(8):e43021. doi: 10.1371/journal.pone.0043021 (PMC3418234; doi:10.1371/journal.pone.0043021)
Supplement: Table S3 — Concentrations of activated fatty acids (acylcarnitines), sterols, and plasma glycerophospholipids composition in patients with phenylketonuria (PKU) and healthy controls. (DOC) [file pone.0043021.s003.doc]

**Table S3**

| **Metabolite** | **PKU**  **median (range)** | **Controls**  **median (range)** | **p-value** |
| --- | --- | --- | --- |
| **Activated fatty acids** |  |  |  |
| Total acylcarnitines [µmol/l] | 6.33 (3.3-9.8) | 8.40 (5.8-13.3) | **p<0.001** |
| Carnitine free (C0) [µmol/l] | 21.5 (13-42) | 31.6 (21-44) | **p<0.01** |
| Acetylcarnitine (C2) [µmol/l] | 10.4 (6-17) | 13.1 (10-19) | **p<0.001** |
| Propionylcarnitine (C3) [µmol/l] | 0.81 (0.4-1.3) | 1.28 (0.5-2.2) | **p<0.001** |
| Malonylcarnitine (C3DC) [µmol/l] | 0.04 (0-0.07) | 0.07 (0-0.2) | 0.065 |
| Butyrylcarnitine (C4) [µmol/l] | 0.15 (0.1-0.3) | 0.17 (0-0.4) | 0.258 |
| 3-Hydroxy-Butyryl-Carnitine (C4-OH) [µmol/l] | 0.06 (0-0.21) | 0.10 (0.04-0.29) | **p<0.01** |
| Isovalerylcarnitine (C5) [µmol/l] | 0.08 (0-0.2) | 0.14 (0-0.2) | **p<0.01** |
| Tiglylcarnitine (C5:1) [µmol/l] | 0.02 (0-0.05) | 0 (0-0.06) | 0.861 |
| 2-Hydroxyisovalerylcarnitine, 3S-Hydroxy-3-methyl-glutarylcarnitine (C5 OH+HMG) [µmol/l] | 0.16 (0.04-0.31) | 0.25 (0.05-0.53) | **p<0.05** |
| Hexanoylcarnitine (C6) [µmol/l] | 0.04 (0 – 0.12) | 0.07 (0-0.11) | **p<0.05** |
| Octanoylcarnitine (C8) [µmol/l] | 0.03 (0-0.1) | 0.09 (0-0.2) | **p<0.01** |
| Octenoylcarnitine (C8:1) [µmol/l] | 0.07 (0-0.4) | 0.15 (0.02-0.32) | 0.053 |
| Decanoylcarnitine (C10) [µmol/l] | 0.07 (0-0.14) | 0.11 (0-0.38) | 0.19 |
| Decenoylcarnitine (C10:1) [µmol/l] | 0.03 (0-0.13) | 0.07 (0-0.2) | **p<0.05** |
| Methylmalonylcarnitine (MMA) [µmol/l] | 0.49 (0.05-0.93) | 0.58 (0.26-0.90) | 0.282 |
| Glutarylcarnitine [µmol/l] | 0.03 (0-0.08) | 0.04 (0-0.13) | **p<0.05** |
| Dodecanoylcarnitine (C12) [µmol/l] | 0.04 (0-0.12) | 0.07 (0-0.16) | 0.10 |
| Tetradecanoylcarnitine (C14) [µmol/l] | 0.10 (0.03-0.2) | 0.11 (0-0.26) | 0.348 |
| Tetradecenoylcarnitine (C14:1) [µmol/l] | 0.03 (0-0.08) | 0.07 (0-0.19) | **p<0.05** |
| 3-Hydroxy-tetradecanoylcarnitine (C14OH) [µmol/l] | 0 (0-0.03) | 0 (0-0.04) | 0.972 |
| Hexadecanoylcarnitine (C16) [µmol/l] | 0.85 (0.39-1.47) | 1.05 (0.69-1.63) | 0.092 |
| 3-Hydroxy-hexadecanoylcarnitine (C16OH) [µmol/l] | 0 (0-0.04) | 0 (0-0.08) | 0.73 |
| Hexadecenoylcarnitine (C16:1) [µmol/l] | 0.04 (0-0.1) | 0.07 (0-0.13) | **p<0.05** |
| 3-Hydroxy-hexadecenoylcarnitine (C16:1OH) [µmol/l] | 0.04 (0-0.1) | 0.04 (0-0.11) | 0.525 |
| Octadecanoylcarnitine (C18) [µmol/l] | 0.52 (0.22-0.88) | 0.76 (0.4-1.4) | **p<0.001** |
| 3-Hydroxy-octadecanoylcarnitine (C18OH) [µmol/l] | 0 (0-0.05) | 0 (0 -0.02) | **p<0.001** |
| Octadecenoylcarnitine (C18:1) [µmol/l] | 0.66 (0.2-1.3) | 1.04 (0.7-1.5) | **p<0.001** |
| Trans,trans-9,12-octadecadienoic acid (C18:2) [µmol/l] | 0.16 (0.08-0.29) | 0.22 (0.08-0.31) | **p<0.05** |
| **Sterols in serum** |  |  |  |
| Brassicasterol (BRT) [mg/l] | 0.65 (0.26 - 1.28) | 0.61 (0.32 - 1.37) | 0.964 |
| Campesterol (CAT) [mg/l] | 3.00 (1.15 - 5.37) | 3.01 (2.42 - 7.62) | 0.497 |
| Stigmasterol (STT) [mg/l] | 0.47 (0.25 - 0.8) | 0.48 (0.36 – 1.06) | 0.221 |
| Beta-Sitosterol (SIT) [mg/l] | 1.60 (0.7 – 3.35) | 1.76 (1.33 – 5.39) | 0.441 |
| Lanosterol free (LAF) [mg/l] | 0.07 (0.05 – 0.11) | 0.06 (0.05 – 0.08) | 0.390 |
| Cholesterol (CHT) [mg/l] | 1720 (1422 – 1930) | 1713 (1639 – 1846) | 0.821 |
| Desmosterol/Zymosterol/7-DHC-Sterol (DEZY7DHCT) [mg/l] | 2.93 (2.35 - 3.89) | 2.83 (2.58 – 3.24) | 0.556 |
| CAT/CHT x 1000 (Absorption marker) | 1.74 (0.81 – 3.71) | 1.82 (1.41 – 4.19) | 0.497 |
| LAF/CHT x 1000 (Synthesis marker) | 0.04 (0.02 - 0.08) | 0.04 (0.03 – 0.05) | 0.189 |
| **Plasma glycerophospholipid composition [mg/l]** |  |  |  |
| C14:0 | 6.4 (3.1 – 10.4) | 4.5 (1.8 – 8.5) | 0.231 |
| C14:1 | 0.4 (0 – 0.8) | 0.25 (0.0 – 0.7) | 0.505 |
| C15:1 | 0.3 (0 – 1.0) | 0.6 (0.3 – 0.9) | 0.226 |
| C16:0 | 308.8 (249.4 – 409.6) | 301.3 (223.0 – 352.0) | 0.869 |
| C16:1n-7 | 9.6 (3.4 – 20.8) | 7.0 (5.1 – 16.3) | 0.215 |
| C17:0 | 3.7 (2.5 – 5.8) | 3.7 (2.6 – 4.8) | 0.901 |
| C18:0 | 167.4 (136.5 – 220.8) | 150.4 (126.1 – 185.6) | 0.283 |
| C18:1n-9 | 145 (118.5 – 311.5) | 131.7 (112.1 – 165.2) | 0.409 |
| C18:1n-7 | 15.8 (13.6 – 19.0) | 15.4 (11.9 – 19.3) | 0.509 |
| C18:2n-6 | 234 (164.8 – 337.8) | 256.1 (186.2 – 328.1) | 0.620 |
| C18:3n-6 | 2.1 (1.0 – 5.1) | 1.1 (0.6-2.4) | **<0.01** |
| C18:3n-3 | 3.4 (1.1 – 11.5) | 3.6 (1.5 – 7.40) | 0.741 |
| C20:1n-9 | 1.7 ( 1.3 – 2.0) | 1.6 (1.3 – 2.4) | 0.834 |
| C20:2n-6 | 3.7 (2.6 – 5.6) | 3.9 (3.0 – 4.6) | 0.535 |
| C20:3n-9 | 2.7 (0.2 – 44.8) | 2.8 (1.6 – 3.6) | 0.741 |
| C20:3n-6 | 43.3 (33.0 – 60.0) | 36.2 (30.0 – 52.8) | 0.069 |
| C20:4n-6 | 121.6 (89.2 – 162.3) | 118.3 (101.6 – 131.3) | 0.869 |
| C20:3n-3 | 0.6 (0.3 -1.1) | 0.65 (0.4 – 0.8) | 1.000 |
| C20:5n-3 | 7.7 (1.1 – 15.3) | 7.7 (4.9 – 29.40) | 0.901 |
| C22:4n-6 | 5.9 (4.1 – 12.5) | 4.8 (3.6 – 6.0) | 0.214 |
| C22:5n-6 | 5.0 (2.5 – 12.8) | 4.6 (2.7 – 5.6) | 0.408 |
| C22:5n-3 | 12.3 (6.3 – 18.7) | 11.8 (8.1 – 16.3) | 0.535 |
| C22:6n-3 | 28.4 (18.6 – 36.6) | 33.6 (23.6 – 43.7) | 0.186 |
| Total | 1130.7 (954.6 – 1489.8) | 1100 (8867.5 – 1303.1) | 0.620 |
